# Discipline in Sanatoria : The Grumbles of Working-Class Patients

**Published:** 1914-12-05

**Authors:** 


*To the Editor of* The Hospital.

### Discipline in Sanatoria: The Grumbles of Working-Class Patients.

*To the Editor of* The Hospital.

Sir,—The question of discipline in sanatoria is an ever-present one, and the problem of maintaining it there presents the same difficulty as in all convalescent institutions, only more so.

The sanatorium patient comes for a long period—anything from three to six months—and because he feels his strength coming back to him long before the treatment has done its permanent work, he chafes at the idea of having to undergo it after he has felt its benefit. To him the extra weeks are unnecessary and even wasteful, from a wage-earning standpoint, and it is very necessary to make him see that his cure, to be permanent, must be extended.

The main cause for grumbling in sanatoria, and there is always grumbling, is because the patient gets tired of the treatment, and as he must give a reason for discontent or for discharging himself, he almost always puts it down to the food.

It is a curious fact that the food so often forms the basis of discontent in sanatoria, and yet this turns out to be always unfounded. The food is the main treatment, and perhaps bound to be a little monotonous, being restricted to the most wholesome and simple kinds. Because of this it is very necessary to shuffle the menu well, so that meals are not anticipated. As the patient stays for such a long time this becomes doubly important. The grumbler is always with us, and he is bound to have his effect upon others. When he cannot be reformed he must be weeded out.

Patients' work is an endless source of difficulty too. The average working-class patient does not take kindly to it. First of all, he has a rooted belief in rest and idleness for his complaint. This comes about chiefly from the fact that rest is the first thing his doctor has told him to seek, but the patient does not reason with himself that the conditions under which he has been working must naturally cease: the crowded, ill-lit, ill-ventilated workshop, where he spends some fourteen hours daily. (These are the places from whence are drawn the patients with which the writer has to do.) Such a patient finds it difficult to see that the graduated work given at the sanatorium, done under ideal conditions, is not given from motives of economy of labour, to get work done on the cheap, but solely for the patients' good. They argue that if they can work here they can work at home for wages. The patient has to be taught to view this part of the treatment in the right light.

Discipline in the way of smoking, gambling, and restrictions outside the grounds has to be enforced, and regulations laid down with regard to them must be adhered to, and penalties imposed. Half-measures in these matters will always be useless. Patients must be instructed to co-operate in their treatment by keeping the regulations made for their sole good; they must help towards their own recovery. Tact and experience will always show where to allow a little rope. The too-tight rein will defeat its own ends by causing underhand methods on their part.—Yours, etc.,

A Sanatorium Officer of Ten Years' Experience.

